# Effect of various levels of standardized ileal digestible branched-chain amino acids on lactating sow and litter performance

**DOI:** 10.1093/tas/txae148

**Published:** 2024-10-14

**Authors:** Dalton C Humphrey, Keith D Haydon, Laura L Greiner

**Affiliations:** Department of Animal Science, Iowa State University, Ames, IA 50011, USA; CJ America – Bio, Fort Dodge, IA 50501, USA; Department of Animal Science, Iowa State University, Ames, IA 50011, USA

**Keywords:** branched-chain amino acids, isoleucine, lactation, leucine, sow, valine

## Abstract

Three hundred and sixty sows were used to investigate the effect of various dietary branched-chain amino acids (BCAA) levels on sow lactation and piglet growth performance. On day 112 ± 1.4 of gestation, sows were blocked by the parity group (P1, P2, P3+) and randomly assigned to 1 of 6 dietary treatments containing various levels of standardized ileal digestible (SID) Leu, Ile, and Val. The experimental diets were formulated to the desired levels of BCAA by replacing cornstarch in a basal diet with l-leucine, l-isoleucine, and l-valine. Dietary BCAA levels relative to SID Lys were 114% or 180% for Leu, 56% or 64% for Ile, and 64% or 120% for Val. Diets were formulated to be isocaloric (3.23 Mcal ME/kg) and met or exceeded all other NRC (2012) essential amino acid and vitamin and mineral recommendations. Sow body weight (BW) and backfat thickness were measured at the time of entry into the farrowing room and at weaning. Piglet litter weights were recorded after cross-fostering and weaning to calculate the litter growth rate. Data were analyzed using generalized linear mixed models with fixed effects of dietary treatment and parity group and a random effect of lactation group. The models were fit using R (v4.4.1; R Core Team, 2024). The sow and her litter were the experimental unit, and results were considered significant if *P* < 0.05. On average, sows nursed their litters for 21.3 d (*P* = 0.998). The mean parity by treatment ranged from 3.8 to 3.9 (*P* = 0.999). After farrowing, the mean sow BW was 220 kg with a range between treatments of 216 to 222 kg (*P* = 0.523). On average, sows gained 2.3% of their BW (*P* = 0.740) with an average daily feed intake of 8.74 kg/d (*P* = 0.903). As expected, sow Leu, Ile, and Val intakes were different across treatments (*P* ≤ 0.001) and corresponded to the varying dietary levels of BCAA. Sows entered farrowing with an average backfat thickness of 11.50 mm (*P* = 0.919) and lost 6.5% backfat through lactation (*P* = 0.880). Sows started the trial with an average of 14.1 piglets/sow (*P* = 0.967) and weaned 12.7 piglets/sow (*P* = 0.995) with a piglet ADG of 0.22 kg/d (*P* = 0.280) and a daily litter growth rate of 2.90 kg/d (*P* = 0.547). In conclusion, there was no evidence of an effect of the various leucine, isoleucine, and valine levels evaluated in this study on lactating sow and piglet performance.

## Teaser

At the levels of intake observed, the combinations of leucine, isoleucine, and valine evaluated did not impact lactating sow or piglet performance.

## Introduction

Lactation is the most metabolically demanding period in the reproductive cycle of a sow, requiring increased amounts of energy, fatty acids, glucose, and amino acids compared to other stages of production to support milk production. Among these nutrients, the branched-chain amino acids (BCAA) – leucine, isoleucine, and valine – are taken up by the mammary gland in greater quantities than exported in milk protein, suggesting they may serve additional metabolic functions within the mammary gland beyond their direct use for milk protein synthesis, such as oxidation, nonessential amino acid (NEAA) synthesis, or synthesis of enzymes and structural proteins (Linzell et al., 1969; Trottier 1997; Krogh et al., 2017). Previous in vitro research has shown that BCAA are extensively catabolized in porcine mammary tissue, resulting in the production of NEAA, such as glutamine and aspartate, both of which are abundant NEAA in sow milk (Li et al., 2009). Furthermore, porcine mammary tissue lacks the enzymes required to convert arginine, ornithine, or proline into glutamine (O’Quinn et al., 2002). Together this suggests that BCAA serve an important role in NEAA synthesis for milk protein. Additionally, the end-products of BCAA catabolism can enter the Krebs cycle and contribute to ATP production, and the ketogenic products of Leu and Ile catabolism can be utilized for de novo fatty acid synthesis (Brosnan and Brosnan, 2006). Therefore, aside from their role as constituents in milk protein, BCAA likely serve additional metabolic roles within the mammary gland to support milk production.

Given that BCAA are dietary essential amino acids in swine, ensuring adequate intake of these amino acids during lactation is imperative to maximize milk yield and avoid excessive tissue mobilization, which is known to have negative consequences on subsequent reproductive performance in the sow. According to the NRC (2012), the standardized ileal digestible (SID) Leu, Ile, and Val requirements in lactating sows (parity 2+) nursing 11.5 piglets with a daily litter gain of 2.65 kg/d are 55.2, 27.2, and 41.6 g/d, respectively. These levels correspond to a SID Leu:Lys, Ile:Lys, and Val:Lys of 113%, 56%, and 85%, respectively. However, while there has been extensive research investigating the Val requirement of lactating sows, the responses have been inconsistent. As reviewed by Holen et al. (2022), some studies have reported improved litter growth performance with increasing total Val:Lys to greater than 120%, while other studies have concluded no benefit to increasing SID Val:Lys from 55% to 136%. In contrast, there is limited data on the Leu and Ile requirements of lactating sows (Haught and Speer, 1977; Rousselow et al., 1979; Richert et al., 1997a).

A complicating factor in determining BCAA requirements is their metabolic interdependence. Due to common enzymes in their catabolic pathway, excess intake of one BCAA will increase the catabolism of all 3 BCAA, which can cause inadequate availability of the BCAA not provided in excess and is associated with the BCAA antagonism that has been described in multiple species (D’Mello, 2003). Furthermore, BCAA share transporters at the blood–brain barrier with other large neutral amino acids (LNAA), such as Trp, Tyr, and Phe, which are precursors of serotonin, dopamine, and norepinephrine, respectively. Therefore, competitive inhibition of LNAA transport by BCAA and subsequent alterations in neurotransmitter biosynthesis has been proposed to contribute to the adverse effects of high levels of dietary BCAA (Cemin et al., 2019). Together, this suggests that the variation in the response of lactating sows to dietary Val concentration may be due to differences in the dietary Leu and Ile levels across experiments.

Recent research in nursery pigs has demonstrated that interactions between BCAA can significantly affect growth performance and efficiency. For example, increasing dietary Leu depressed feed intake when Val levels were marginal, while feed intake was maintained with elevated Leu when Val was at or above NRC (2012) recommendations. Furthermore, increasing both Leu and Ile in the diet reduced growth rate and resulted in poorer feed efficiency compared to increasing either amino acid alone (Humphrey et al., 2023). Similarly, excess Leu has been associated with depressed growth performance in grow–finish pigs, which was primarily attributed to imbalances amongst the BCAA and with LNAA (Cemin et al., 2019; Kwon et al., 2019). However, there is limited research investigating whether similar relationships exist in lactating sows.

A previous study by Moser et al. (2000) evaluated the effects of BCAA on lactating sows using a 2 × 2×2 factorial design with total Leu at 1.57% or 1.97% (Leu:Lys 174% or 219%), total Ile at 0.98% or 1.08% (Ile:Lys 76 or 120%), and total Val at 0.80% or 1.20% (Val:Lys 89% or 133%). While no interactions between BCAA were observed, increasing Val improved litter weight gain, litter weaning weight, and increased sow backfat loss. Similarly, Richert et al. (1997a) reported increased litter weight gain when Val was increased from 0.72% to 1.07% (Val:Lys 80% to 119%) or Ile from 0.50% to 1.20% (Ile:Lys 56% to 133%) at a constant Leu level (Leu:Lys 150%), but no interactions between Val and Ile. However, in both studies, the dietary Leu levels were well above NRC (2012) requirements, which may have masked potential interactions or benefits that could have resulted from lower levels of Leu. Therefore, the objective of this study was to evaluate the effects of varying combinations of SID Leu, Ile, and Val levels on lactating sow and piglet performance. It was hypothesized that increasing SID Val would improve litter growth, but to differing extents based on the SID Leu and Ile levels in the diet; high levels of both SID Leu and Ile would compromise performance; and high levels of SID Leu in combination with low SID Val would reduce sow feed intake.

## Materials and Methods

### General

All experimental protocols adhered to guidelines for the ethical and humane use of animals for research according to the Guide for the Care and Use of Agricultural Animals in Research and Teaching (FASS, 2010) and were approved by the Institutional Animal Care and Use Committee at Iowa State University (IACUC 23-051).

### Animals

The study was conducted from April through June 2023 at a midwestern commercial sow research facility. A total of 360 primiparous and multiparous sows (PIC Camborough, PIC Genus, Hendersonville, TN [*n* = 319]; Genesus F1 Yorkshire × Landrace [*n* = 41], Genesus Inc., Winnipeg, Manitoba) bred to terminal line boars (PIC 800, PIC Genus, Hendersonville, TN), were evaluated across 5 lactation groups. The sows were porcine reproductive and respiratory syndrome stable. Prior to entering the farrowing room, sows were fed a standard gestation diet containing 3.10 Mcal ME/kg and 0.61% SID Lys according to body condition score (BCS; 4.1, 3.4, and 2.9 kg for BCS 2, 3, and 4, respectively). Upon entering the farrowing room, sows were randomly assigned, within the parity group (P1 [*n* = 70], P2 [*n* = 38], P3+ [*n* = 252]), to 1 of 6 dietary treatments containing various SID Leu:Lys, Ile:Lys, and Val:Lys levels.

### Experimental Diets

The experimental diets were formulated to various SID Leu, Ile, and Val levels by replacing cornstarch in a basal diet with l-Leu, l-Ile, and l-Val (Table 1). The levels of SID Leu, Ile, and Val expressed relative to Lys were 114% or 180%, 56% or 64%, and 64% or 120%, respectively. The lowest SID Leu level was set according to the NRC (2012) recommendation for lactating sows, while the highest level was chosen to reflect the upper range of what is achievable in practical diet formulation when incorporating corn co-products, such as distiller’s dried grains with solubles. For SID Ile, the lowest level aligned with the NRC (2012) recommendation, whereas the highest level was selected based on the upper limit evaluated in Humphrey et al. (2023). The lowest SID Val level was set below the NRC (2012) recommendation and aimed to reflect the lower end of the proposed requirement in the literature (Strathe et al., 2016; Greiner et al., 2019), while the highest level exceeded NRC (2012) recommendations and approached the upper range of the proposed requirement reported in the literature (Richert et al., 1996, 1997a, 1997b; Moser et al., 2000; Xu et al., 2017).

**Table 1.** Ingredient and calculated nutrient composition of experimental diets (as-fed basis)

		Dietary treatment					
	SID^1^ Leu:Lys, %	180	180	180	180	114	114
	SID Ile:Lys, %	64	64	56	56	64	64
	SID Val:Lys, %	064	120	64	120	64	120
Ingredient	SID TBCAA^2^:Lys, %	308	364	300	356	242	298
Corn		69.08	69.08	69.08	69.08	69.08	69.08
Soybean meal 46.5% CP		23.83	23.83	23.83	23.83	23.83	23.83
Corn starch		0.63	0.00	0.73	0.10	1.37	0.74
Calcium carbonate		1.14	1.14	1.14	1.14	1.14	1.14
Monocalcium phosphate		1.03	1.03	1.03	1.03	1.03	1.03
Fat AV blend		1.00	1.00	1.00	1.00	1.00	1.00
Sodium chloride		0.50	0.50	0.50	0.50	0.50	0.50
Other feed additives^3^		0.45	0.45	0.45	0.45	0.45	0.45
l-Lysine HCl		0.44	0.44	0.44	0.44	0.44	0.44
ThrPro^4^		0.31	0.31	0.31	0.31	0.31	0.31
HMTBa^5^		0.20	0.20	0.20	0.20	0.20	0.20
VTM premix with phytase^6^^,^^7^		0.15	0.15	0.15	0.15	0.15	0.15
Choline chloride		0.12	0.12	0.12	0.12	0.12	0.12
l-Histidine HCl		0.11	0.11	0.11	0.11	0.11	0.11
l-Tryptophan		0.07	0.07	0.07	0.07	0.07	0.07
25-Hydroxyvitamin D_3_		0.03	0.03	0.03	0.03	0.03	0.03
l-Valine		0.03	0.65	0.03	0.65	0.03	0.65
l-Isoleucine		0.10	0.10	0.00	0.00	0.10	0.10
l-Leucine		0.79	0.79	0.79	0.79	0.05	0.05
Total		100.00	100.00	100.00	100.00	100.00	100.00
Calculated composition							
ME, Mcal/kg		3.23	3.23	3.23	3.23	3.23	3.23
Crude protein, %		16.50	16.02	16.56	16.08	17.02	16.54
Calcium, %		0.69	0.69	0.69	0.69	0.69	0.69
Available phosphorus, %		0.43	0.43	0.43	0.43	0.43	0.43
Total Lys, %		1.22	1.22	1.22	1.22	1.22	1.22
Total Ile, %		0.80	0.80	0.70	0.70	0.80	0.80
Total Leu, %		2.17	2.17	2.17	2.17	1.45	1.45
Total Val, %		0.82	1.44	0.82	1.44	0.82	1.44
SID Lys, %		1.10	1.10	1.10	1.10	1.10	1.10
SID Ile, %		0.70	0.70	0.62	0.62	0.70	0.70
SID Leu, %		1.98	1.98	1.98	1.98	1.25	1.25
SID Val, %		0.70	1.32	0.70	1.32	0.70	1.32
SID TBCAA, %		3.38	4.00	3.30	3.92	2.65	3.27
SID His:Lys		0.40	0.40	0.40	0.40	0.40	0.40
SID Ile:Lys		0.64	0.64	0.56	0.56	0.64	0.64
SID Leu:Lys		1.80	1.80	1.80	1.80	1.14	1.14
SID Met+Cys:Lys		0.57	0.57	0.57	0.57	0.57	0.57
SID Phe:Lys		0.61	0.61	0.61	0.61	0.61	0.61
SID Tyr:Lys		0.29	0.29	0.29	0.29	0.29	0.29
SID Thr:Lys		0.66	0.66	0.66	0.66	0.66	0.66
SID Trp:Lys		0.22	0.22	0.22	0.22	0.22	0.22
SID Val:Lys		0.64	1.20	0.64	1.20	0.64	1.20

^1^Standardized ileal digestible.

^2^Total branched-chain amino acids.

^3^Included 0.40% Vigilex and 0.05% Promote Diffusion.

^4^Threonine biomass product (CJ America—Bio) provided 80% l-threonine.

^5^HMTBa; Methionine source. 88% 2-hydroxy,4-methylthio butanoic acid.

^6^Vitamin trace mineral premix; provided 9,011 IU vitamin A, 2,254 IU Vitamin D, 60 IU vitamin E, 3.8 mg vitamin K, 7.1 mg riboflavin, 39.9 mg niacin, 2.2 mg thiamin, 1.7 mg folic acid, 0.2 mg biotin, 20 mg Cu (copper sulfate and basic copper chloride), 0.7 mg I (ethylenediamine dihydroiodide), 100 mg Fe (ferrous sulfate), 50.1 mg Mn (manganous oxide), 0.3 mg Se (sodium selenite and selenomethionine hydroxy analogue), and 120 mg Zn (zinc sulfate) per kg of the diet.

^7^Phytase provided 978 FTU per kg of feed, assuming 0.15% available phosphorus release.

Constraints at the farm limited the number of BCAA combinations tested to 6, which restricted the ability to construct a full-factorial design; therefore, the combinations tested were strategically chosen as those that were expected to elicit the most extreme and contrasting response based on the BCAA relationships reported in Humphrey et al. (2023). Specifically, 2 diets were formulated to the highest SID Ile:Lys (56%) and Leu:Lys (180%) levels and either the low or high SID Val:Lys (64% or 120%). Additionally, 2 diets were formulated at the low SID Ile:Lys level, the high SID Leu:Lys level, and either the high or low SID Val:Lys level. The last 2 diets included the highest SID Ile:Lys level (64%), the lowest SID Leu:Lys level (114%), and either the higher or low SID Val:Lys level. The 2 BCAA combinations consisting of the lowest levels of Leu and Ile and either the high or low level of Val were excluded from the design based on the expectation that the response to these combinations would be in the flat portion of the response surface (Humphrey et al., 2023).

Standardized ileal digestible Lys (1.10%) and all other essential amino acids relative to Lys were held constant across the diets. Additionally, the diets were formulated to be isocaloric (3.23 Mcal ME/kg) and met or exceeded all other NRC (2012) essential amino acid and vitamin and mineral requirements. The diets were manufactured at a local feed mill, and feed samples were collected weekly and stored at −20 °C until further analysis.

### Lactation Management and Feeding

Sows were moved into the farrowing room at 112 ± 1.4 d of gestation and were fed their allocated dietary treatments from the time of entry until weaning. Sows were housed in standard farrowing stalls (1.5 × 2.1 m) in an environmentally controlled farrowing room (18 to 30 °C). Sows farrowed at 117 ± 1.4 d of gestation, and piglets were cross-fostered, within dietary treatment, within 24 h after birth.

Prior to farrowing, sows were fed 2.18 kg/d of their assigned lactation diet. Postpartum, sows were fed 4.35 kg on day 1, 6.53 kg on day 2, 8.70 kg on day 3, and then allowed ad libitum feed for the remainder of the lactation period. Feed was hand-delivered to feed hoppers twice daily, with excess feed removed and weighed back as needed. Sows and piglets had ad libitum access to water throughout lactation.

### Sow and Litter Criteria

Sows were weighed at the time of entry into the farrowing room and at weaning. Sow 48h post-farrowing body weight (BW) was calculated using the following empirical equation: postfarrow weight, kg  =  [(d 112 gestation weight, kg) × 0.98) - 20.81 (R2 = 0.93)] (Greiner, unpublished data). The equation for estimating sow post-farrowing BW was developed using sows of a similar weight range and production capacity as the sows in the current study. In addition, sow backfat (BF) at the last rib was measured via ultrasound (Tecnoscan SF-1, Tecnovet, Barcelona, Spain) at entry into the farrowing room and at weaning.

Piglet litter weights were recorded within 24 h of birth and at weaning. Cross-fostering was conducted, within treatment, within 24 h of birth to equalize litter size across treatments. Cross-fostering was done according to the farm’s standard operating procedure, where the biggest piglets from sows with excess piglets were moved to sows with open functional teats. The starting litter weight for the study was the litter weight after cross-fostering. On approximately day 3 of lactation, fall-behind piglets were identified, based on loss of body condition and inability to thrive, and moved to sows that were not in the study. The fall-behind piglets were considered morbidities in subsequent calculations. Piglet mortalities and morbidities were recorded, and the weight and nursing days of the removed pigs were back-calculated into the litter growth rate according to the following equation: [(total litter wean weight-total starting litter weight+removal weights)/(number of piglets weaned ×lactation length+days removals nursed)]. Piglet removal rate was calculated as the number of mortalities plus morbidities divided by litter size after cross-fostering. Mortalities that occurred prior to cross-fostering or litter weights were not included in litter growth or removal rate calculations.

### Sample Collection and Analysis

#### Diet analysis

Weekly feed samples were pooled on an equal weight basis and the composite samples were ground through a 1-mm screen using a Wiley Mill (Variable Speed Digital ED-5 Wiley Mill; Thomas Scientific, Swedesboro, NJ). Ground samples were submitted to the University of Missouri Agricultural Experimental Station Laboratories (Columbia, MO) for complete amino acid profiling using cation-exchange chromatography coupled with post-column ninhydrin derivatization and quantification (method 982.30 E and 988.15; AOAC, 2006). The gross energy of feed samples was also measured at Iowa State University using an isoperibolic bomb calorimeter (model 6200; Parr Instruments Co., Moline, IL).

#### Blood sample collection and plasma analysis

At the time of weaning, approximately 1 h after piglet and feed removal, a blood sample was collected via jugular venipuncture from 10 or 11 sows per treatment (2 or 3 sows per lactation group). Within a lactation group, sows for blood sample collection were chosen to minimize the differences in lactation length. The blood samples were stored on ice until centrifugation at 2,500 G for 10 min at 4 °C. Plasma samples were aliquoted and stored at −80 °C until further analysis. Plasma samples were submitted to the University of Missouri Agriculture Experimental Station Laboratories (Columbia, MO) for plasma urea and free amino acid analysis via high-performance liquid chromatography.

### Statistical Analysis

Data were analyzed in R (v4.4.1; R Core Team, 2024) using generalized linear mixed models with fixed effects of dietary treatment and parity group (P1, P2, P3+) and a random effect of lactation group. The interaction between dietary treatment and the parity group was tested and excluded from all models due to lack of significance (*P* ≥ 0.208). Additionally, because the Genesus F1 sows were only represented in the parity 3+ group, the genetic line was not included in the model. The models for sow feed intake, BW, BF, plasma amino acids, and piglet growth were fit assuming a normal distribution using the lmer function of the lme4 package (v1.1.35.3; Bates et al., 2015). The models for sow parity, lactation length, litter counts, and piglet removals were fit using the glmer function of the lme4 package, assuming a Poisson distribution for parity, lactation length, and litter counts and a binomial distribution for piglet removals.

For models assuming a normal distribution, outlier detection was conducted on the Studentized Residuals, where residuals greater than 3 standard deviations from the mean were considered statistical outliers and excluded from the analysis. The assumptions of all models were verified through residual diagnostic plots created with the check_model function of the performance package (v0.12.0; Lüdecke et al., 2021). The sow and her litter were the experimental unit, and results were considered significant if *P* ≤ 0.05.

## Results

The analyzed total amino acid concentrations of the diets (Table 2) were consistent with calculated values based on normal variation (Cromwell et al., 1999). Specifically, the BCAA levels were elevated or reduced in each diet in accordance with the dietary treatment design, while all other amino acids remained similar across diets; therefore, subsequent results and discussion will be based on the calculated SID BCAA levels.

**Table 2.** Analyzed nutrient composition of experimental diets (as-fed basis)

		Dietary treatment					
	SID^1^ Leu:Lys, %	180	180	180	180	114	114
	SID Ile:Lys, %	64	64	56	56	64	64
	SID Val:Lys, %	64	120	64	120	64	120
Item	SID TBCAA^2^:Lys, %	308	364	300	356	242	298
Gross energy, Mcal/kg		3.86	3.88	3.85	3.86	3.86	3.88
Crude protein, %		16.97	18.28	16.57	17.47	16.56	17.18
Total amino acids, %							
Aspartic acid		1.52	1.68	1.46	1.66	1.51	1.49
Threonine		0.74	0.84	0.74	0.86	0.79	0.79
Serine		0.63	0.69	0.62	0.69	0.65	0.66
Glutamic acid		2.84	3.06	2.71	3.01	2.78	2.72
Proline		1.03	1.07	0.99	1.07	1.01	0.98
Glycine		0.65	0.70	0.62	0.69	0.63	0.62
Alanine		0.84	0.88	0.80	0.87	0.83	0.80
Cysteine		0.26	0.29	0.27	0.28	0.28	0.25
Valine		0.81	1.30	0.77	1.41	0.79	1.28
Methionine		0.22	0.24	0.22	0.23	0.23	0.22
Isoleucine		0.78	0.85	0.68	0.76	0.77	0.73
Leucine		2.07	2.15	2.04	2.14	1.48	1.41
Tyrosine		0.52	0.56	0.50	0.55	0.52	0.51
Phenylalanine		0.79	0.85	0.75	0.84	0.78	0.76
Lysine		1.16	1.27	1.12	1.28	1.15	1.17
Histidine		0.49	0.53	0.50	0.52	0.47	0.49
Arginine		0.96	1.05	0.91	1.03	0.94	0.93
Tryptophan		0.23	0.23	0.23	0.23	0.23	0.24

^1^Standardized ileal digestible.

^2^Total branched-chain amino acids.

Sows nursed their litters for an average of 21.3 d (Table 3; *P* = 0.998). The average parity across treatments was 3.8 (*P* = 0.999). After farrowing, sow BW was similar across treatments (*P *= 0.523), with a range between treatments of 216 to 222 kg. Through lactation, sows gained, on average, 2.3% of their BW (*P* = 0.740) with an average daily feed intake (ADFI) of 8.73 kg/d (*P* = 0.903); however, sows lost 6.5% BF (*P* = 0.880). Lysine intake was similar across treatments and averaged 96 g/d (*P* = 0.902). As expected, Leu, Ile, Val, and total branched-chain amino acid (TBCAA) intake differed between treatments (*P* < 0.001). Leucine intake was higher in sows fed diets containing SID Leu:Lys of 180% (172 to 175 g/d) than diets containing SID Leu:Lys of 114% (107 g/d). Similarly, Ile intake was higher in sows fed diets containing SID Ile:Lys of 64% (61 to 62 g/d) compared to diets containing SID Ile:Lys of 56% (53 to 54 g/d). Furthermore, Val intake was higher in sows fed diets containing SID Val:Lys of 120% (115 to 118 g/d) compared to diets containing SID Val:Lys 64% (60 to 61 g/d). Consequentially, TBCAA intake mirrored dietary TBCAA concentrations and ranged from 228 to 353 g/d.

**Table 3.** Effect of dietary branched-chain amino acid levels on sow lactation performance

		Dietary treatment								
	SID^1^ Leu:Lys, %	180	180	180	180	114	114			
	SID Ile:Lys, %	64	64	56	56	64	64			
	SID Val:Lys, %	64	120	64	120	64	120		*P*-value	
Item	SID TBCAA^2^:Lys, %	308	364	300	356	242	298	SEM	Trt	Parity
Average parity		3.8	3.8	3.9	3.8	3.9	3.8	0.28	0.999	—
Parity 1, *n*		13	13	10	11	12	11	—	—	—
Parity 2, *n*		6	6	6	7	6	7	—	—	—
Parity 3+, *n*		41	41	44	42	42	42	—	—	—
Total born, *n*/sow		17.1	17.1	16.3	16.9	17.1	16.7	0.57	0.855	0.268
Lactation length, d		21.4	21.1	21.1	21.4	21.3	21.3	0.64	0.998	0.766
ADFI^3^, kg/d		8.80	8.77	8.83	8.62	8.69	8.71	0.215	0.903	<0.001
SID Lys intake, g/d		97	96	97	95	96	96	2.4	0.902	<0.001
SID Leu intake, g/d		175^a^	174^a^	175^a^	172^a^	107^b^	107^b^	3.7	<0.001	<0.001
SID Ile intake, g/d		62^a^	62^a^	54^b^	53^b^	61^a^	61^a^	1.4	<0.001	<0.001
SID Val intake, g/d		61^b^	118^a^	61^b^	115^a^	60^b^	116^a^	2.1	<0.001	<0.001
SID TBCAA intake, g/d		298^b^	353^a^	291^b^	340^a^	228^c^	285^b^	7.3	<0.001	<0.001
48h BW^4^, kg		221	221	216	220	220	222	3.4	0.523	<0.001
Weaning BW, kg		225	224	223	225	225	227	3.1	0.943	<0.001
BW change, kg		3.9	2.9	7.4	4.3	4.5	4.4	2.56	0.744	<0.001
BW change, %		2.1	1.5	3.6	2.1	2.3	2.1	1.14	0.740	<0.001
Entry BF^5^, mm		11.7	11.4	11.3	11.4	11.4	11.8	0.52	0.919	0.001
Weaning BF, mm		11.1	10.5	10.6	10.5	10.7	11.1	0.45	0.778	0.164
BF change, mm		−0.8	−0.9	−0.8	−0.9	−0.7	−0.8	0.21	0.962	<0.001
BF change, %		−5.2	−7.0	−6.1	−7.6	−6.5	−6.3	1.84	0.880	<0.001

^1^Standardized ileal digestible.

^2^Total branched-chain amino acids.

^3^Average daily feed intake.

^4^Body weight.

^5^Backfat.

^a,b,c^Within a row, means without a common superscript differ (*P* ≤ 0.05).

After cross-fostering, sows started the trial with an average of 14.1 piglets/sow (Table 4; *P* = 0.967) with an average litter weight of 21.0 kg (*P* = 0.305), which resulted in a similar average piglet start weight across treatments (*P* = 0.800). Sows weaned an average of 12.7 piglets/sow (*P* = 0.995) with a piglet average daily gain of 0.22 kg/d (*P* = 0.280) and a daily litter growth rate of 2.90 kg/d (*P* = 0.457). Although the piglet removal rate was numerically highest in litters of sows fed the diet containing 180% SID Leu:Lys, 64% SID Ile:Lys, and 64% SID Val:Lys, there was little evidence for a statistical difference in piglet removals between treatments (*P* = 0.076).

**Table 4.** Effect of dietary branched-chain amino acid level on litter performance

		Dietary treatment								
	SID^1^ Leu:Lys, %	180	180	180	180	114	114			
	SID Ile:Lys, %	64	64	56	56	64	64			
	SID Val:Lys, %	64	120	64	120	64	120		*P*-value	
Item	SID TBCAA^2^:Lys, %	308	364	300	356	242	298	SEM	Trt	Parity
Number started^3^, *n*/sow		14.5	14.1	14.2	14.1	14.0	13.8	0.52	0.967	0.309
Litter start weight, kg		21.50	21.12	21.17	21.11	20.65	20.19	0.475	0.305	0.642
Average piglet start weight, kg		1.51	1.51	1.52	1.49	148	1.46	0.030	0.800	<0.001
Number weaned, *n*/sow		12.6	12.7	12.9	12.7	12.7	12.5	0.50	0.995	0.174
Litter weaning weight, kg		79.13	81.90	80.19	80.82	79.68	79.36	1.967	0.810	<0.001
Average piglet weaning weight, kg		6.29	6.53	6.35	6.39	6.26	6.43	0.142	0.441	<0.001
Litter ADG^4^, kg/d		2.83	2.99	2.92	2.91	2.87	2.88	0.069	0.457	<0.001
Piglet ADG, kg/d		0.22	0.23	0.22	0.22	0.22	0.23	0.005	0.280	<0.001
Removals, %^5^		12.65	9.92	9.06	9.57	8.86	9.54	1.207	0.076	0.017

^1^Standardized ileal digestible.

^2^Total branched-chain amino acids.

^3^Litter size after cross-fostering, which occurred within treatment, within 24 h of birth.

^4^Average daily gain.

^5^Removals = mortalities + morbidities.

Plasma Val concentration followed dietary SID Val levels and intake, where plasma Val was higher in sows fed diets containing a SID Val:Lys of 120% compared to sows fed SID Val:Lys of 64% (Table 5; *P* < 0.001). Plasma Ile concentration was higher in sows fed the diet containing 114% SID Leu:Lys, 64% Ile:Lys, and 120% Val:Lys compared to sows fed either diet with a SID Ile:Lys of 56% or the diet containing 180% SID Leu:Lys, 64% Ile:Lys, and 120% Val:Lys, while the sows fed the other 2 treatments had intermediate plasma Ile levels (*P* < 0.001). Plasma Leu concentrations were higher in sows fed the diets containing a SID Leu of 180%, Val:Lys of 64%, and Ile:Lys of 56 or 64% compared to sows fed the diet containing 114% SID Leu:Lys, 64% Ile:Lys, and 64% Val:Lys, while the sows fed the other 3 diets had intermediate plasma Leu levels (*P* = 0.007). As a result, there were differences between treatments in plasma TBCAA concentration (*P* < 0.001). Interestingly, sows fed the diet containing the second lowest TBCAA concentration (SID TBCAA:Lys 298%) had the highest plasma TBCAA concentration compared to sows fed the diet with the lowest TBCAA level (SID TBCAA:Lys 242%).

**Table 5.** Effect of dietary branched-chain amino acid level on lactating sow plasma urea and amino acid concentrations

		Dietary treatment								
	SID^1^ Leu:Lys, %	180	180	180	180	114	114			
	SID Ile:Lys, %	64	64	56	56	64	64			
	SID Val:Lys, %	64	120	64	120	64	120		*P*-value	
Item	SID TBCAA^2^:Lys, %	308	364	300	356	242	298	SEM	Trt	Parity
Urea, µM		4,988.4	5,107.3	5,491.9	5,056.7	5,101.0	5,187.3	307.92	0.868	0.483
Proteogenic amino acids, µM										
Alanine		407.4^ab^	352.5^b^	396.9^b^	417.5^ab^	407.0^ab^	523.3^a^	39.03	0.006	0.446
Arginine		154.7	110.0	133.7	122.4	123.4	149.6	20.25	0.347	0.889
Asparagine		80.3	55.1	82.0	67.3	66.5	87.5	10.91	0.058	0.406
Aspartic acid		10.2	9.0	10.4	10.0	10.0	10.7	1.05	0.865	0.195
Cystine		48.4	46.7	47.1	45.6	46.7	50.2	2.23	0.517	0.311
Glutamic acid		123.7	119.9	112.6	122.4	126.2	129.0	16.06	0.981	0.119
Glutamine		403.6	360.6	381.2	378.4	353.7	404.1	25.79	0.436	0.638
Glycine		922.3	704.7	860.4	802.2	843.1	881.6	66.18	0.206	0.639
Histidine		83.1	78.9	81.2	71.8	78.6	80.5	6.38	0.829	0.146
Isoleucine		91.5^ab^	84.7^b^	68.9^b^	71.8^b^	98.4^ab^	123.6^a^	9.83	0.001	0.522
Leucine		295.5^a^	232.5^ab^	287.0^a^	275.6^ab^	182.5^b^	211.6^ab^	28.65	0.007	0.844
Lysine		274.9	176.0	228.0	207.9	228.3	258.1	38.99	0.300	0.747
Methionine		44.9	35.7	42.5	36.6	38.0	47.9	4.46	0.163	0.286
Phenylalanine		67.3	70.0	71.4	59.5	60.7	74.8	6.50	0.360	0.656
Proline		241.0	192.2	245.4	234.7	222.4	272.6	25.47	0.093	0.925
Serine		104.1	88.9	95.8	94.9	89.6	108.4	6.75	0.128	0.812
Threonine		250.9	184.2	259.3	222.9	244.1	268.0	33.42	0.363	0.841
Tryptophan		48.8	49.4	52.3	48.9	49.1	54.4	4.56	0.818	0.828
Tyrosine		71.2	60.0	72.6	59.5	66.0	77.0	8.90	0.437	0.882
Valine		169.0^b^	353.7^a^	152.0^b^	426.9^a^	178.2^b^	444.9^a^	45.17	<0.001	0.422
TBCAA		187.9^bc^	219.2^abc^	172.0^bc^	252.2^ab^	155.8^c^	305.7^a^	27.76	<0.001	0.844
Non-proteogenic amino acids, µM										
Carnosine		23.2	25.8	23.9	22.5	27.7	24.1	1.37	0.077	0.757
Citrulline		96.9	87.0	96.3	92.1	95.9	100.6	9.32	0.910	0.055
Cystathionine/allocystathionine		2.0	2.9	3.3	2.4	3.1	2.8	0.77	0.657	0.005
Hydroxyproline		41.1	40.4	46.1	41.6	45.8	47.4	3.70	0.411	0.001
Ornithine		69.0	52.9	66.8	63.0	59.6	71.0	11.13	0.612	0.352
Phosphoserine		17.0	12.8	16.7	14.4	16.7	16.6	1.35	0.127	0.948
Sarcosine		74.0^b^	69.2^b^	81.3^ab^	80.6^ab^	97.7^a^	98.2^a^	8.05	0.001	0.798
Taurine		49.3	57.2	54.9	52.3	51.3	51.2	6.04	0.914	0.764
1-Methyl histidine		10.5^ab^	8.6^b^	11.3^a^	10.5^ab^	9.3^ab^	10.4^ab^	0.65	0.027	0.034
3-Methyl histidine		25.2	25.5	27.9	26.1	24.5	24.8	2.75	0.875	0.004
𝛼-Amino adipic acid		87.4	80.8	98.2	90.1	92.5	98.4	11.95	0.848	0.406
𝛼-Amino n-butyric acid		25.5	28.1	27.2	24.5	27.9	29.6	2.91	0.638	0.007
𝛽-Alanine		16.0	16.7	15.4	15.7	16.9	16.2	0.83	0.716	0.111

^1^Standardized ileal digestible.

^2^Total branched-chain amino acids.

## Discussion

The Val requirement of lactating sows has been a topic of extensive research; however, the results reported across the literature have been inconsistent. While some authors have reported improved sow and piglet performance at total Val:Lys levels greater than 120%, others have observed no benefit from increasing SID Val:Lys from 55% to 136% (Holen et al., 2022). In contrast, little work has been conducted to understand the Leu and Ile requirements of lactating sows. Haught and Speer (1977) evaluated the total Ile requirement of lactating sows and observed a quadratic response of piglet weight gain, which was maximized at 0.37% Ile (total Ile:Lys 67%). Rousselow et al. (1979) suggested the Leu requirement of lactating sows to be 0.46% (total Leu:Lys 79%) based on metabolic data but observed no improvements in piglet growth. Subsequently, Richert et al. (1997a) reported linear improvements in litter weight gain with increased total Ile:Lys from 56 to 133% at either 80% or 119% total Val:Lys and a constant Leu level. However, the NRC (2012) did not report the sources used to establish their estimated Ile and Leu requirements for lactating sows.

The discrepancies in the optimal BCAA levels reported across the literature may, in part, be related to the interconnectedness of BCAA metabolism, which is unique in that the first 2 steps in the catabolic pathway are common amongst the 3 amino acids, including reversible transamination by branched-chain amino acid transferase and irreversible decarboxylation by the branched-chain α-keto acid dehydrogenase complex (BCKD; Brosnan and Brosnan, 2006). Due to these shared catabolic enzymes, excess of one BCAA will stimulate catabolism of all 3, which may result in limited availability of the BCAA not provided in excess. Furthermore, α-ketoisocaproate, the α-keto acid of Leu catabolism, is the most potent allosteric inhibitor of BCKD kinase, which inhibits BCKD activity (Brosnan and Brosnan, 2006). Therefore, excess Leu consumption may elicit an antagonistic response more readily in practical swine diets when Val or Ile are supplied near the sow’s requirements. Thus, a potential source of variation between studies in the response to dietary Val may be the accompanying Leu and Ile levels in the diets.

In the present study, lactating sow and piglet performance was not impacted by the various combinations of BCAA levels evaluated, providing little evidence that the response to one BCAA may depend on the levels of the other 2 in the diet. Feed intake in the current study was higher than anticipated but was within current feed intake averages based on information from the genetic supplier (PIC North America, 2024). The high level of feed intake resulted in BCAA and Lys intakes greater than those observed in previous BCAA research in lactating sows. The SID Lys level in the diets was set according to the estimated requirement of the sows (Greiner et al., 2020) based on the current average sow lactation feed intake and litter growth performance at the research farm prior to the current study. The dietary BCAA levels were subsequently set relative to Lys to achieve the desired SID Leu:Lys, Ile:Lys, and Val:Lys. However, the observed sow ADFI in this study was approximately 20% greater than anticipated, which resulted in sows consuming SID Lys above the estimated requirement and SID Leu, Ile, and Val intake ranging from 107 to 175, 53 to 62, and 60 to 118 g/d, respectively. Similarly, Strathe et al. (2016) observed no impact of increasing SID Val:Lys from 76% to 97%, corresponding to a SID Val intake of 37 to 42 g/d and SID Ile and Leu intakes of approximately 52 and 29 g/d, respectively. Greiner et al. (2019) also reported no benefit to increasing SID Val:Lys from 55 to 100%, with SID Val intakes of 31 to 50 g/d and SID Ile intake of 30 g/d. Furthermore, Devi et al. (2015) reported no impacts of increasing SID Val:Lys from 80% to 85% (37 to 42 g/d SID Val intake) in primiparous sows but did not report the Leu or Ile concentrations of the diets. In contrast, Xu et al. (2017) increased SID Val:Lys from 63% to 123% and reported a linear improvement in piglet growth rate; however, this was also associated with increased Lys intake due to the linear response of ADFI reported by the authors.

In earlier studies that directly evaluated interactions between BCAA in lactating sows, increasing total Val intake from 48 to 64 g/d improved litter weight gain regardless of total Leu and Ile intake; however, there were no benefits observed when total Leu intake increased from 87 to 103 g/d or when total Ile intake increased from 40 to 59 g/d (Moser et al., 2000). Furthermore, Richert et al. (1997a) reported increased litter weight gain with increased total Val intake from 45 to 65 g/d and increased total Ile intake from 31 to 74 g/d, but there were no interactions between Val and Ile. Therefore, BCAA intakes at the lower levels of Leu, Ile, and Val evaluated in the present study may have been greater than the sows’ quantitative BCAA requirements, resulting in a lack of response to further increasing levels in the diet and may have mitigated potential issues associated with BCAA antagonism.

Other researchers have investigated increasing TBCAA levels in sow lactation diets at a constant Leu:Ile:Val. Rezaei et al. (2022) fed sows diets containing 3.20%, 4.74%, or 6.39% TBCAA and reported increased piglet growth rate with increasing TBCAA and reduced sow BW loss in sows fed 6.39% TBCAA. Similarly, Ma et al. (2020) increased dietary TBCAA concentration from 2.85% to 3.24% at a constant Leu:Ile:Val and observed litter ADG and weights at weaning. The SID TBCAA levels in the current study ranged from 2.65% to 4.00%; however, because Leu:Ile:Val was not constant across diets, it is difficult to compare these results to those reported previously.

It has been previously observed that excess Leu in the diet negatively impacts ADFI in nursery pigs when Val levels are below requirements but not when Val is sufficient (Humphrey et al., 2023); therefore, if dietary Val was sufficient in all diets within the current study, the expectation would be that Leu would not impact ADFI in the sow. The lack of response of sow ADFI to varying levels of BCAA in the diet observed in the current study is in agreement with previously conducted BCAA research in sows (Richert et al., 1996, 1997a, 1997b; Carter et al., 2000; Moser et al., 2000; Gaines et al., 2006; Devi et al., 2015; Strathe et al., 2016; Greiner et al., 2019; Rezaei et al., 2022). Additionally, the lack of response of ADFI to increased Leu at differing Val levels may also indicate that there are inherent differences in the mechanisms regulating feed intake in lactating sows compared to growing animals, which may mitigate the anorexigenic response to high dietary Leu, but further work is needed to differentiate between these 2 potential explanations.

In general, sow plasma Leu, Ile, and Val increased with their respective concentration in the diet; however, the differences in circulating BCAA were not as definitive as those observed with BCAA intake. For example, while plasma Val concentrations were higher in sows fed diets containing a SID Val:Lys of 120% compared to 64%, plasma concentrations of Leu and Ile were more variable compared to SID Leu and Ile intakes. Additionally, plasma TBCAA was highest in sows fed the diet with the second lowest SID TBCAA concentration, which could indicate reduced BCAA catabolism compared to diets with higher TBCAA levels but may also indicate reduced utilization of BCAA by tissues. The inconsistencies between BCAA intake and circulating levels in the current study may provide biological evidence of interactions between BCAA. In support of this observation, Moser et al. (2000) reported tendencies for interactions between Ile and Leu for plasma Ile levels and between Val and Leu for plasma Val. However, drawing conclusions based on venous concentrations of amino acids is limited because amino acid delivery and use by tissues depends on arterial blood concentrations, rate of uptake, and blood flow. Thus, further research is warranted to elucidate the differences in plasma BCAA levels observed in this study.

In conclusion, there is no evidence that, at the level of intake observed in this study, the various BCAA levels evaluated impact lactating sow or piglet performance. However, further work is needed to validate these conclusions at lower levels of Leu, Ile, and Val intake. Furthermore, additional work may be warranted to evaluate if BCAA imbalances impact subsequent or long-term reproductive performance in sows.

## Abbreviations

ADFI: average daily feed intake
ADG: average daily gain
BCAA: branched-chain amino acids
BCS: body condition score
BF: backfat
BW: body weight
LNAA: large neutral amino acids
NEAA: nonessential amino acids
SID: standardized ileal digestible
TBCAA: total branched-chain amino acids
